# Assessment of Vascular Changes in Patients after Pars Plana Vitrectomy Surgery Due to Macula-Off Rhegmatogenous Retinal Detachment

**DOI:** 10.3390/jcm10215054

**Published:** 2021-10-28

**Authors:** Anita Lyssek-Boroń, Adam Wylęgała, Katarzyna Krysik, Dominika Janiszewska-Bil, Edward Wylęgała, Beniamin Oskar Grabarek, Dariusz Dobrowolski

**Affiliations:** 1 Department of Ophthalmology, Faculty of Medicine in Zabrze, University of Technology, 41-800 Zabrze, Poland; kkrysik@gmail.com (K.K.); dominika.bjaniszewska@gmail.com (D.J.-B.); 2 Trauma Centre, Department of Ophthalmology, St. Barbara Hospital, 41-200 Sosnowiec, Poland; dardobmd@wp.pl; 3 Health Promotion and Obesity Management Unit, Department of Pathophysiology, Faculty of Medical Sciences in Katowice, Medical University of Silesia, 40-055 Katowice, Poland; adam.wylegala@gmail.com; 4 Chair and Clinical Department of Ophthalmology, Division of Medical Science in Zabrze, Medical University of Silesia in Katowice, 40-760 Katowice, Poland; ewylegala@sum.edu.pl; 5 Department of Ophthalmology, District Railway Hospital, 40-760 Katowice, Poland; 6 Department of Histology, Cytophysiology and Embryology, Faculty of Medicine, University of Technology in Katowice, 41-800 Zabrze, Poland; bgrabarek7@gmail.com

**Keywords:** macula-off rhegmatogenous detachment, optical coherence tomography angiography, superficial perifoveal capillary plexus, retinal deep perifoveal capillary plexus

## Abstract

The aim of this study was to investigate the changes in the retinal capillary plexuses in patients after pars plana vitrectomy (PPV), which is used for the treatment of rhegmatogenous retinal detachment (RRD). In this study, we included the results of 114 patients who underwent PPV after total retinal detachment (RRD; retinal detachment group). It should be kept in mind that to qualify for the study group, there was a condition that retinal detachment be only present in one eye, allowing the fellow healthy eye to be used for the control group, and the study, therefore, did not include cases where retinal detachment occurred binocularly. Optical coherence tomography (OCT) and OCT-A images were taken at 9 ± 2 months (median 10 months) after the surgery, with the study conducted in the years 2017–2019. OCT was used to examine the external limiting membrane (ELM), central macular thickness (CMT) and retinal nerve fiber layer (RNFL), while OCT-angiography (OCT-A) was used to examine the extent of the foveal avascular zone (FAZ) in the deep and superficial capillary plexuses. Changes in the FAZ area of the superficial plexus (SCP) between the study and control groups were analyzed over 346 ± 50 days. In our study, we observed changes in the FAZ area between the RRD and control groups in the SCP (203.65 ± 31.69 μm^2^ vs. 215.30 ± 35.82 μm^2^; *p* = 0.28733) and DCP (284.79 ± 35.82 µm^2^ vs. 336.84 ± 32.23 µm^2^; *p* = 0.00924). Changes in the RNFL thickness between the study and control groups over 346 ± 50 days were as follows: 90.15 μm vs. 82.44 μm; *p* = 0.19773. Disruption of the external limiting membrane was observed in 78.95% (90 eyes) of the study group. In the control group, it was undamaged, and no integrity disorder was observed. In the RRD, changes occurred in the FAZ of both the SCP and the DCP, which reduced the extent of this zone, an effect that was more pronounced in DCPs. A better understanding of the anatomical and hemodynamic changes taking place in the retina after macula-off RRD might be helpful in answering the question as to why BCVA in these cases is “only” or “as much as” from 0.4 to 0.1, namely, that it might be related to changes in the neurosensory retina after macular peeling.

## 1. Introduction

In cases of rhegmatogenous retinal detachment (RRD) with macular plexus, retinal detachment does not always guarantee that anatomical success will equate to functional success, which is highly expected from the patient. In such cases, pars plana vitrectomy is an efficient procedure, which involves removal of the internal limiting membrane (ILM) to relax the central retina and prevent formation of the secondary epiretinal membrane (ERM) [1,2]. The ideal operative outcome is when the anatomical success of retinal reattachment due to RRD is equal to or greater than 90% of the treated cases [3,4].

One of the first reports of visual recurrence after surgery for retinal macular detachment was reported by Burton et al. [5]. It was observed that in 46 out of 87 patients (53%; *p* < 0.05) who had been operated on up to 9 days from the onset of symptoms, the visual acuity after the procedure was in the range of 20/20–20/50. However, when the procedure was performed between 10 and 19 days after symptom onset, the percentage of patients with the same level of visual acuity after the procedure was only 34% (*p* < 0.05), and for surgery following an even longer period after symptom onset, only 29% (*p* < 0.05) [5]. Park et al. [6] distinguished six factors determining visual acuity after surgery. These include ellipsoidal integrity impairment (β = 0.167; *p* < 0.001), Henle fiber layer and outer nuclear layer (HFL + ONL)/photoreceptor layer ratio (β = 0.199; *p* < 0.001), photoreceptor outer segment length (β = −0.020; *p* < 0.001), ratio of photoreceptor inner segment length to photoreceptor outer segment length (β = 0.047; *p* = 0.005), ratio of photoreceptor layer thickness between the RD and other eye (β = −0.126; *p* = 0.018) and the ratio of the length of the outer segment of photoreceptors between the RD and the other eye (β = −0.425; *p* < 0.001) [6].

In turn, Geiger et al. [7] found a visual acuity of at least 20/40 in 81 out of 131 patients (61.8%) receiving surgery 6 months after retinal detachment. These authors emphasized that patients who had better VA logMAR before surgery had better post-operative VA (*p* < 0.05), and that the time from retinal detachment to surgery did not significantly affect post-operative VA [7].

Optical coherence tomography (OCT) is a method that exploits the phenomenon of low-coherence interferometry. As a consequence, it is possible to visualize eye structures in vivo. OCT has become a key component in the clinical evaluation of the cornea and anterior segment of the eye. OCT is used in the diagnosis of cystic swelling of the macula, epiretinal membrane (ERM) and retinal folds [8,9] as well as changes within the external limiting membrane (ELM), which is formed from the combination of photoreceptors and Müller cells [10]. The possibility to measure the central macular thickness (CMT) and the thickness of the retinal nerve fiber layer (RNFL) provides additional prognostic information [11]. Optical coherence tomography angiography (OCT-A) is an examination that may provide further information on the changes occurring in the highly important process of macular vascularization after its reattachment. The retinal vascular plexus of healthy subjects is formed by a superficial capillary plexus (SCP) located in the ganglion cell layer and nerve fiber layer and a deep capillary plexus (DCP) located in the inner nuclear layers [12,13]. OCT-A has been used in the diagnosis and monitoring of treatment effects in the case of macular degeneration, retinal vein obstruction, diabetic retinopathy, posterior uveitis and optic neuropathies [14,15,16,17]. McKay et al. [18] used the OCT-A technique to compare the vessel density (VD) and FAZ in the eyes of a group of patients, in which there was one affected eye that had undergone macula-off RRD and the fellow eye for each patient. VD was significantly lower in the RRD eyes compared with the fellow eyes (*p* < 0.05). It was also reported that visual acuity after surgery was poorer for RRD eyes with decreased VD in the DCP compared with other control eyes without VD [18].

Additionally, Bonfiglio et al. [19] reported a reduction in vascular density in the superficial and deep retinal plexuses in RRD eyes after PPV compared with other healthy eyes [19].

Woo et al. [20], in turn, found that, in a group of 34 RRD patients who underwent PPV with gas tamponade, the superficial and deep FAZ areas were significantly larger in the macula-off group (superficial: 0.374 ± 0.112 mm^2^; deep: 0.702 ± 0.193 mm^2^) than in the macula-on group (superficial: 0.282 ± 0.105 mm^2^; deep: 0.543 ± 0.114 mm^2^) following surgery. Post-treatment FAZ enlargement may result from ischemic damage to the retinal capillary plexus in the fovea [20].

In the superficial plexus, the vessels are linear with a centripetal pattern; in the deep plexus, the vessels have a concentric distribution with vertical interconnections. The foveal capillary plexus forms a ring at the margin of the fovea, producing a capillary-free region called the fovea avascular zone (FAZ) [21]. Macular peeling is a surgical technique used in diseases of the retina, which can include primary or secondary diabetic retinopathy, retinal detachment, holes, macular edema or foveal retinoschisis. The technique is based on surgical removal of the preretinal tissue or internal limiting membrane (ILM) in the macula. The term ILM was first used in 1845 by Pacini to describe the boundary between the retina and the vitreous. It is a periodic acid–Schiff (PAS)-positive basement membrane within which astrocytes and the end feet of Müller cells can be distinguished [22,23]. Complications that may occur after macula peeling include cataract progression, intraocular pressure increase, visual field defects, retinal tears, retinal detachment, vitreous hemorrhage, ocular hypotony, dislocation of the intraocular lens in pseudophakic eyes, macular phototoxicity, RPE changes and endophthalmitis [24,25].

Our study aimed to evaluate the changes occurring in superficial and deep perifoveal capillary-free zones in patients after undergoing pars plana vitrectomy (PPV) with macular peeling surgery in which the reason for operation was rhegmatogenous retinal detachment with macular plexus.

## 2. Materials and Methods

### 2.1. Patients

The research was approved by the Research Ethics Committee of the Silesian Medical Chamber in Katowice, Poland (Resolution Number ŚIL/KB/100p/17). Patient consent to review their medical records was not required by the bioethical committee due to the retrospective nature of this study and because patient information was sufficiently anonymized. The study was conducted in the years 2017–2019.

In this study, we included the results for 114 patients who underwent PPV after total retinal detachment (RRD; retinal detachment group). It should be kept in mind that to qualify for the study group, there was a condition that retinal detachment be only present in one eye, allowing the fellow healthy eye to be used for the control group, and the study therefore did not include cases where retinal detachment occurred binocularly. OCT-A images were obtained by scanning using a DRI OCT Topcon Triton (Top-con, Tokyo, Japan) with Net 6 imaging by a single trained technician. This device utilizes swept-source technology with a central wavelength of 1050 nm and can capture more than 100,000 scans/second [10]. FAZ was measured using ImageNet (Topcon Medical Systems Inc., Oakland, NJ, USA). The ELM was classified as disrupted when there were signs of any discontinuity, fusion or thickening in the scan OCT. This retrospective study included patients who underwent successful pars plana vitrectomy or combined phacovitrectomy with ILM peeling for idiopathic macula-off rhegmatogenous retinal detachment at St. Barbara Regional Specialist Hospital in Poland. OCT-A was conducted at the Department of Ophthalmology, District Railway Hospital Katowice in Poland.

The exclusion criteria for all participants were as follows: history of trauma; high myopia with an axial length of more than 26.5 mm; other retinal diseases; history of uveitis; diabetic retinopathy; optic disc abnormality; optic nerve disorder; previous intraocular surgery; low-quality OCT-A images; duration of retinal detachment from the uveal membrane longer than 2 months from the appearance of prodromal symptoms.

### 2.2. Surgical Procedure

All patients signed to indicate their informed consent before undergoing any surgical procedure. All eyes underwent 23-gauge, 3-port pars plana vitrectomy performed by one vitreoretinal surgeon (A.L.-B.) with the same technique, using the same vitreoretinal machine (Constellation, Alcon, Fort Worth, TX, USA). All phakic patients underwent PPV with phacoemulsification and intraocular lens implantation. First, posterior vitreous detachment was confirmed using Brilliant Blue staining, unless core vitrectomy with increased pressure beginning at the optic disc was performed. Then, peripheral vitrectomy was performed. The next step was the injection of 0.1 mL solution of Brilliant Blue over the retinal surface for 1 min. The internal limiting membrane (ILM) was removed with forceps. The ILM was peeled in a circular fashion with a 2-disc diameter around the macula. Afterwards, fluid–air exchange and 360-degree endolaser photocoagulation were performed. Then, air–gas or air–silicone oil exchange was performed. Oil endotamponade was removed in all patients after 1–1.5 months after PPV; 5000-centistoke silicone oil was used. A corticosteroid and an antibiotic were applied after surgery for four weeks (fludrocortisone acetate + gramicidin + neomycinum, Polfa Warszawa, Warszawa, Poland) according to the recommendation.

### 2.3. Statistical Analysis

To analyze the results of the first observation, the average RNFL thickness, 4-quadrant RNFL thickness, central macular thickness and FAZ in the superficial and deep plexuses were calculated and displayed, with ELM structures being analyzed. Statistical analysis was performed using STATISTICA 13.3 software (StatSoft, Cracow, Poland) with a statistical significance threshold of *p* < 0.05.

The normality of the data was assessed using the Shapiro–Wilk test. In order to determine whether the differences between the groups were statistically significant, Student’s *t*-test was performed for independent groups (*p* < 0.05).

## 3. Results

We analyzed the OCT results of 228 eyes and the OCT-A results of 114 patients (63 women and 51 men) after an observation period of 346 ± 50 days. The mean age of the group was 63.06 ± 8.96 years. Before surgery, cataracts were removed in 38 (33.33%) eyes that qualified for PPV (RRD group) surgery and in 15 healthy eyes (13.16%). In the remaining eyes, however, an artificial lens was implanted during the PPV procedure. Two types of endotamponade were used depending on the intraoperative evaluation, i.e., silicone oil (removed 1–1.5 months after PPV) in 80 eyes and 20% SF6 in 34 eyes [26,27]. Each patient underwent a complete ophthalmologic examination, including best corrected visual acuity testing using Snellen charts, slit lamp examination and OCT-A, 9 ± 2 months (median 10 months; range 6–17 months) after the operation. The characteristics of the vitrectomized eyes are summarized in Table 1.

**Figure 1:**
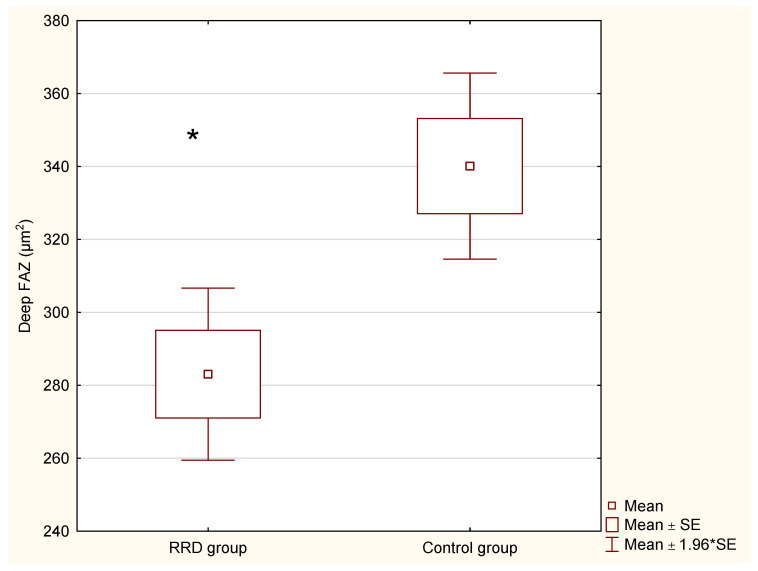
Changes in the deep vascular plexus of the FAZ in the RRD group and in the control group (*p* < 0.05). RRD—rhegmatogenous retinal detachment group; FAZ—fovea avascular zone; * statistically significant differences in comparison to control (*p* < 0.05).

In our study, we observed changes in the FAZ area between the RRD and control groups in the SCP (203.65 ± 31.69 μm^2^ vs. 215.30 ± 35.82 μm^2^; *p* = 0.28733; non-statistically significant) and the DCP (284.79 ± 35.82 µm^2^ vs. 336.84 ± 32.23 µm^2^; *p* = 0.00924; Figure 1).

The mean RNFL increased slightly in the post-treatment group only in the temporal quadrant, with the increase being statistically insignificant (*p* = 0.104).

**Figure 2:**
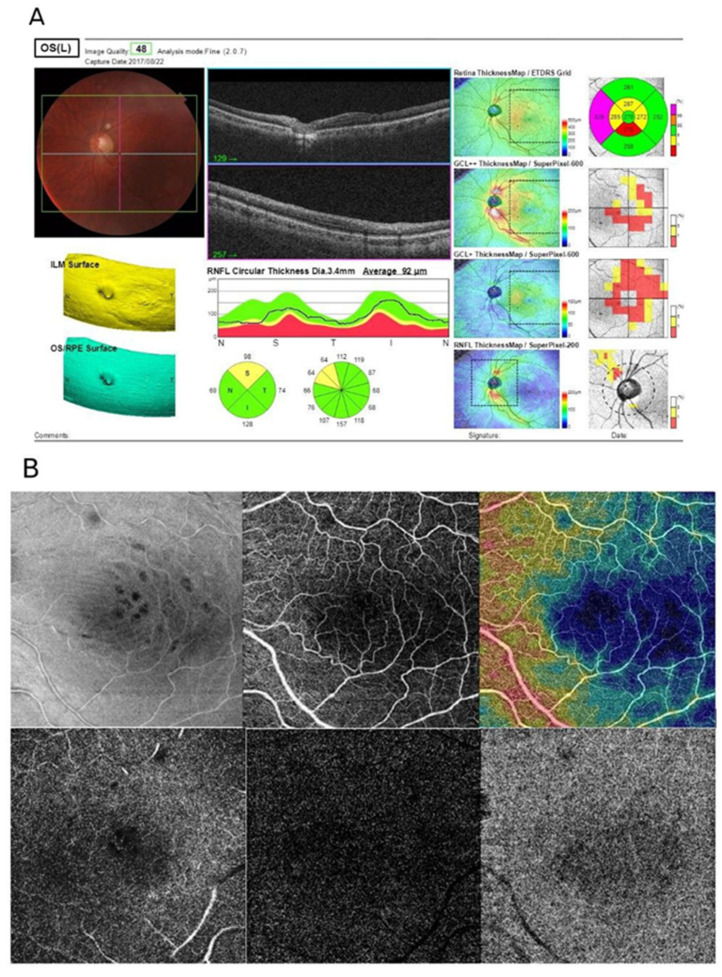
Comprehensive report of a patient who underwent PPV for RRD. (**A**) the OCT-A scans; (**B**) the vascular density map; PPV—pars plana vitrectomy; RRD—rhegmatogenous retinal detachment.

The CMT in both groups exhibited similar values. On the other hand, in 78.95% of cases (90 eyes), the ELM was damaged, while in 50.00% of cases (57 eyes) it remained intact. Although the central macular thickness was normal, there was a visible diffuse edema. Furthermore, the RNFL thickness was below the norm in superior quadrants. The B-scan OCT shows a damaged ELM. The OCT-A scans of the same patient (upper row, left to right) show the en face image, superficial capillary plexus, deep capillary plexus, density map, outer retina and choriocapillaris. The vascular density map shows that the vessels in the temporal segments have lower density, which might result from the lower macular thickness in this region, as shown in the B-scan. Furthermore, the en face image shows the damage occurring to the ELM during PPV (Figure 2).

Changes in the tomography results between the groups are presented in Table 2 and Figure 1. Only differences in the deep FAZ between the RRD group and control group were found to be statistically significant (*p* < 0.05).

## 4. Discussion

Experimental studies conducted on animal and human models demonstrate that apoptosis—the death of photoreceptor cells—occurs 1–3 days after retinal detachment [28,29,30].

Hagimura et al. [31] analyzed macular detachment in terms of macular elevation and changes that take place in an ablated retina. They determined that in the case of a slight elevation of the macula, the ablated retina was thicker, yet it did not exhibit intraretinal abnormalities. In the case of a considerable RRD with macular detachment, there were intraretinal separations of the neurosensory retina and wavy separation of external retina layers. They observed the occurrence of greater impairment of post-operative BCVA in eyes with a greater retinal plexus, despite a brief time of detachment, which suggests irreversible central retina damage [31]. This could explain why 1–2 days after macular detachment, visual acuity after surgical detachment of the retina was not higher than 20/50.

After analysis of our results, we did not determine any increase in CMT in comparison to the control group. We observed that an increased RNFL thickness occurred in the temporal quadrants, yet the increase was not statistically significant. In the remaining three quadrants, the RNFL thickness was slightly lower than in the control group. This result differs from that in currently published reports, which have indicated thinning changes in all four quadrants and, also, differences in the corresponding period in which they were observed (12–24 months) [32,33,34,35]. An increase in RNFL thickness in the temporal quadrants within 6 months after PPV with macular peeling due to ERM was observed [36], yet after this period, thinning occurred. This has been linked to the swelling of nerve fibers stemming from axonal transport disorder, leading to the apoptosis and atrophy of ganglion cells [37,38]. The maintenance of the increase in RNFL thickness in patients after PPV due to macula-off RRD may be linked to (1) intraretinal abnormalities related to macular detachment, (2) the presence of residual fluid related to RPE function disorder and (3) ILM peeling in the macular area.

ELM damage as observed in the OCT provides approximate information on the loss of nuclei of photoreceptor and Müller cells, leading to the loss of photoreceptor function, which results in reduced visual acuity in long-term observations [39]. Post-operative ELM integrity may constitute an important prognostic factor for restoration of the normal distribution of internal and external photoreceptor segments (IS/OS), being linked to enhanced visual acuity [3]. Within our group, in the majority of tested patients, i.e., 90 persons (78.95%), the ELM integrity was disturbed; this BCVA was about 20/50 (0.624 ± 0.22).

In our study, we observed changes in the FAZ area between the RRD and control groups in the SCP (203.65 ± 31.69 μm^2^ vs. 215.30 ± 35.82 μm^2^; *p* = 0.28733; non-statistically significant) and the DCP (284.79 ± 35.82 µm^2^ vs. 336.84 ± 32.23 µm^2^; *p* = 0.00924).

This observation may also confirm the thesis that the shift and deformation of the pit may result in vascular changes [40], and macula-off retinal detachment may lead to changes being intensified in the DCP, caused by intraretinal separations of the neurosensory retina.

Another explanation for this vascular asymmetry may be the performance of ILM peeling, a procedure which, in the case of RRD, is recommended by many experienced vitreoretinal surgeons and is aimed at preventing the development of post-operative epiretinal membranes [1,41]. The ILM is a basement membrane for Müller cells, protecting retina neurons through neurotrophic factor release, which also helps neurons to thrive [41]. It has also been suggested that Müller cells play a role in regulation of the retinal blood flow and angiogenesis [41].

Our results are in line with the observations made by McKay et al. [18], who also reported lower SCP FAZ in the RRD group compared to the control. The opposite was true of the DCP FAZ, as in our study. However, the differences were not found to be statistically significant due to the relatively small size of the groups. At the same time, those authors indicated that the most likely cause of the poorer visual acuity in this group of patients was changes in vessel density (VD) in the DCP [18].

This was also confirmed through studies by Bonfiglio et al. [19], who also showed a lower VD in the RRD group after PPV with gas tamponade compared to the control group [19]. Additionally, Tsen et al. [T15.] showed a statistically significantly smaller area of both the SCP and the DCP in eyes with RRD compared to the control, which is consistent with our observations. In addition, it was noted that in the group of patients who underwent only PPV, they were characterized by higher post-operative vessel density and larger SCP and DCP area in the choroid compared to vitrectomized and scleral buckle eyes [15].

The observations in the study of Wang et al. are also interesting [14], in which macular perfusion changes were assessed in 14 patients (14 eyes) after 23-guage PPV with gas tamponade using OCT-A compared to fellow unaffected eyes. The superficial capillary plexus flow density, deep capillary plexus flow density and choriocapillary plexus flow density were characterized in the RRD group. It should be noted, however, that these investigators found that the FAZ area was significantly larger in the eyes with RRD after the 2-month post-operative period compared to the control eyes [14]. As a consequence, macular hypoxia continued. Furthermore, Woo et al. [20] noted that the superficial and deep FAZ area was significantly larger in the RRD group compared to the control group in a study of 34 patients (34 eyes). The authors suggested that the enlargement of the FAZ area in the RRD group compared to the control group may be due to ischemic damage to the retinal capillary plexus in the fovea [20].

These two observations contradict our results, where the superficial and deep FAZ area was greater in the control group than in the study group. It should be noted, however, that the size of the groups in our study was larger than in the abovementioned studies, which may explain the discrepancy. It should also be remembered that in the studies of Wang et al. [14] and Woo et al. [15], all patients enrolled in PPV underwent gas endotamponade. Meanwhile, in our study, patients received either gas endotamponade (80 patients) or silicone oil (34 patients), which may have had an impact on the obtained results. It should be remembered that the utilization of oil tamponade is not just associated with benefits for the patients but also with side effects. Raczyńska et al. [42] analyzed the influence of silicone tamponade on the ganglion cell complex, engaged in the transmission of visual information from the retina to the brain. They determined that in a group of 57 patients for whom PPV was conducted due to rhegmatogenous retinal detachment (RRD) where silicone oil was utilized, a reduction in the ganglion cell complex was observed; additionally, a deterioration in vision was observed. They also indicated that determining the number of ganglion cells should be the gold standard of determining an improvement or deterioration in vision after eye surgery with silicone oil tamponade [42]. Furthermore, Inoue et al. [43] assessed the influence of perfluorocarbon liquid (PFCL) and silicone oil on human retinal pigment epithelium (RPE) cells and retinal ganglion cells (RCGs), except under in vitro conditions. They observed that PFCL and silicone oil decrease the vitality of RPE cells after 7 days of exposure. Moreover, they also determined that the damage caused by PFCL had a cytotoxic character, and that of silicone oil was mechanical [43], whereas in our research, we did not indicate a cytotoxic effect of oil endotamponade, which may be related to the fact that the silicone oil was removed after a maximum period of 1.5 months in all patients, among other factors. Of course, in light of the abovementioned research [42,43], it seems that a valuable supplement to the research would be the inclusion of a group of patients in whom oil endotamponade or gas would be removed at a later time.

Furthermore, Christou et al. [44] showed a lower VD in SCPs among 23 RRD patients who underwent PPV and applied gas endotamponade compared to the control. The fellow eye was used as a control. It is true that the SCP FAZ in the RRD group was greater than that in the control group, i.e., different than in our study, but the differences were not statistically significant (*p* > 0.05) [44].

Chatziralli et al. [45] assessed changes in the retinal microvasculature in 89 RRD patients who underwent a PPV and gas endotamponade procedure without internal limiting membrane peeling. As a control, they used eyes from healthy volunteers rather than the patient’s healthy eye, as in our study. On the basis of the results from the OCT-A study, they found a statistically significant enlargement of the SCP FAZ and DCP FAZ in the study group after surgery compared to the control (*p* < 0.05). The differences in the scores obtained by Chatziralli et al. [45] and our group may result from the difference in the selection of control groups. Therefore, to address these discrepancies, one more control group should be included in future study [45].

In another study, Chatziralli et al. [46] showed that age, duration of RD, presence of proliferative vitreoretinopathy (PVR), central retinal thickness (CRT) and condition of the ellipsoid zone (EZ) and the ELM should be included among the independent factors affecting visual acuity after PPV surgery in patients with RRD [46].

Informing patients about the factors affecting BCVA will help to better visualize their prognosis after surgery, and this will be helpful in managing the patients. Furthermore, considering the conducted statistical analysis and its results, it would be appropriate to increase the size of the groups. Moreover, an interesting supplement to our research would be the inclusion of a control group comprising healthy volunteers.

Thus, performing ILM peeling in eyes after RRD may result in reduced flow in the capillaries of the internal retina. The possibility for a non-invasive retinal examination provides us with new information on the healing process of this highly important eye structure [46].

The factors that limit our work and results include the retrospective nature of the research presented in this paper. Next, we plan to conduct a prospective study that will include a control group comprising non-peeled eyes. In addition, at a later stage, vessel density analysis should be carried out, and the size of the groups should be increased. Nevertheless, the results obtained, and conclusions drawn from our study, are important, although further research is necessary.

Changes in the FAZ occurred in the study group and were more prevalent in the DCP. After surgical detachment of the retina, not only do anatomical changes take place but, most importantly, the action that is responsible for acquisition of a renewed neurosensory retina with retinal pigment epithelium (RPE) also occurs, resulting in enhanced visual acuity. This depends on numerous factors, the analysis of which has allowed for improved prognostic evaluation of individual cases of RRD in the last decade due to the presence of OCT. This might be related to changes in the neurosensory retina after macular peeling.

## 5. Conclusions

OCT-A is a promising method allowing for non-invasive visualization of the vascular network of the retina, which constitutes a very important component of the repair and nutritional processes in the course of different retinal diseases. A better understanding of the anatomical and hemodynamic changes taking place in the retina after macula-off RRD might be helpful to answer the question as to why the BCVA in these cases is “only” or “as much as” from 0.4 to 0.1.

## Figures and Tables

**Table 1:** Characteristics of RRD group and control group.

Parameter	RRD Group	Control Group
BCVA (logMAR)	0.62 ± 0.22	0.83 ± 0.15
Age (years)	63.07 ± 8.96	
Number of eyes	114	114
Oil tamponade	80 (70.18%)	0
20% SF6 endotamponade	34 (29.82%)	0
Follow-up (days)	346 ± 50	
Pseudophakia before surgery	38 (33.33%)	15 (13.16%)
Axial length	23.41 ± 0.81	22.8 ± 0.21

BCVA—best corrected visual acuity; mean ± standard deviation.

**Table 2:** Comparison of tomographic results between the groups.

Parameter	Group	Mean ± SD	*p* < 0.05
CMT (µm)	RRD	223.55 ± 11.25	*p* = 0.91688
	Control	225.29 ± 12.20	
RNFL	RRD	90.15 ± 4.04	*p* = 0.19773
	Control	82.44 ± 4.26	
Superficial FAZ (μm^2^)	RRD	203.65 ± 31.69	*p* = 0.28733
	Control	215.30 ± 28.52	
Deep FAZ (μm^2^)	RRD	284.79 ± 35.82	*p* = 0.00924
	Control	336.84 ± 32.23	
RNFL	RRD I	100.35 ± 6.56	*p* = 0.39141
	Control I	109.40 ± 8.12	
	RRD S	91.22±7.07	*p* = 0.25636
	Control S	104.20±8.76	
	RRD N	62.65±5.26	*p* = 0.44899
	Control N	69.07±6.52	
	RRD T	79.13±5.39	*p* = 0.63863
	Control T	75.07±6.68	

CMT—central macular thickness; RNFL—retinal nerve fiber layer; FAZ—foveal avascular zone; PPV—pars plana vitrectomy; ELM—external limiting membrane; SD—standard deviation.

## Data Availability

The data used to support the findings of this study are included in the article.
